# Morphometric and physical characteristics distinguishing adult Patagonian lamprey, *Geotria macrostoma* from the pouched lamprey, *Geotria australis*

**DOI:** 10.1371/journal.pone.0250601

**Published:** 2021-05-05

**Authors:** Cindy F. Baker, Carla Riva Rossi, Pamela Quiroga, Emily White, Peter Williams, Jane Kitson, Christopher M. Bice, Claude B. Renaud, Ian Potter, Francisco J. Neira, Claudio Baigún

**Affiliations:** 1 National Institute of Water and Atmospheric Research Ltd, Hamilton, New Zealand; 2 Instituto de Diversidad y Evolución Austral (IDEAus-CONICET), Puerto Madryn, Chubut, Argentina; 3 Kitson Consulting Ltd, Otatara, Invercargill, New Zealand; 4 Inland Waters and Catchment Ecology Program, South Australian Research and Development Institute (SARDI), Aquatic Sciences, Henley Beach, SA, Australia; 5 School of Biological Sciences, The University of Adelaide, Adelaide, SA, Australia; 6 Research and Collections, Canadian Museum of Nature, Ottawa, ON, Canada; 7 Centre for Sustainable Aquatic Ecosystems, Harry Butler Institute, Murdoch University, Murdoch, WA, Australia; 8 Neira Marine Sciences Consulting (Marscco), Blackmans Bay, Tasmania, Australia; 9 Instituto de Investigación e Ingeniería Ambiental (CONICET-UNSAM), San Martín, Buenos Aires, Argentina; Laboratoire de Biologie du Développement de Villefranche-sur-Mer, FRANCE

## Abstract

The pouched lamprey, *Geotria australis* Gray, 1851, has long been considered monotypic in the Geotriidae family with a wide southern temperate distribution across Australasia and South America. Recent studies have provided molecular and morphological evidence for a second *Geotria* species in South America; *Geotria macrostoma* (Burmeister, 1868). The aim of this study was to determine morphometric and physical characteristics of adult *G*. *macrostoma* that further differentiate this re-instated species of Geotriidae from *G*. *australis*. The diagnostic features discriminating immature adult *G*. *macrostoma* from *G*. *australis* when entering fresh water, are distinct differences in dentition, oral papillae and fimbriae counts and differences in coloration. In addition, *G*. *macrostoma* display greater growth of the prebranchial region and oral disc and has a deeper body depth and higher condition factor. All current ecological knowledge of the genus *Geotria* is based on Australasian populations, which may not be applicable to *G*. *macrostoma*. To ensure the conservation and protection of the Patagonian lamprey as a re-identified species, further investigations are needed to understand its life history, biology and ecology throughout its range.

## Introduction

Lampreys are ancient jawless fishes with a lineage dating back around 500 million years [1]. In their review, Docker and Potter [2] reported that lampreys, which have an antitropical distribution, are represented by over 40 species. The majority of those species are restricted to the Northern Hemisphere and allocated to the single family Petromyzontidae, whereas only five are confined to the Southern Hemisphere and allocated to either Mordaciidae (three species) or Geotriidae (two species) [3, 4]. Lampreys display a range of life history strategies. Ten lamprey species are anadromous and feed parasitically in the ocean as juveniles, nine are freshwater residents that also feed parasitically as juveniles, and the large number of remaining species are freshwater residents that are non-parasitic and do not feed as juveniles [2, 4, 5].

Until the study of Riva-Rossi et al. [4], the pouched lamprey, *Geotria australis* Gray, 1851, was the sole recognized species within Geotriidae. It has an anadromous life history characterized by a free-swimming parasitic marine phase, upstream migration and freshwater spawning and larval development [6]. Length-frequency distributions indicate that the larval phase takes between 3 and 4.5 years [7–9]. After completing metamorphosis, the resultant juveniles migrate to the ocean and feed parasitically on fish and marine mammals for 3 to 4 years. Between late summer and early spring, *G*. *australis* returns as adults to fresh water where it spends 14–16 months maturing and then spawning and dying [6, 10–12]. During this protracted spawning run and maturation, the adults do not feed and shrink by up to a third of their body length [10, 12].

The pouched lamprey has a wide southern temperate distribution, documented to inhabit southwestern and southeastern Australia, Tasmania, New Zealand, Chile, Argentina, Falkland (Malvinas) Islands, South Georgia Island (Georgias del Sur) and historical records from Uruguay [13–15]. Recent investigations indicate that this extensive range reflects, in part, an unresolved taxonomy within Geotriidae. Nardi et al. [16] and Riva-Rossi et al. [4] provided genetic and morphological evidence for the presence of a second *Geotria* species in South America. The data of Riva-Rossi et al. [4] indicated that *Geotria* distributed along the south-east coast of South America (from 35°S to 55°S), should be returned to its earliest valid name, *Geotria macrostoma* (Burmeister, 1868). Riva-Rossi et al. [4] termed *G*. *macrostoma* the Argentinian pouched lamprey, but we propose the common name Patagonian lamprey since Patagonia is the geographic region where *G*. *macrostoma* was resurrected and where extant breeding populations are still widespread.

Between 1851 and 1915, researchers postulated that, on the basis of morphological differences, there were eight lamprey genera and 11 species across the Southern Hemisphere, particularly in South America [17–26]. However, Maskell [27] concluded that the variable characters represented different stages of ontogeny and that there are only two Southern Hemisphere genera, i.e. *Mordacia* and *Geotria*. Holly [28] subsequently extended the genera to include the genus *Exomegas* and many authors continued to record *Exomegas* for several decades [29, 30]. Strahan [31] and Potter and Strahan [29] supported the conclusions of Maskell [27] in assigning the four Southern Hemisphere lamprey species to either *Geotria* or *Mordacia* with *Geotria* monotypic. Since the 1950s, there has been a paucity of studies on lampreys in South America and the synonymy of *G*. *australis* continued to be accepted across its range. Although Neira et al. [13] found some distinct morphological differences between the ammocoetes (larvae) of *G*. *australis* from Australasia and Chile versus those from Argentina, no further taxonomic revision was undertaken until Riva-Rossi et al. [4].

The long unresolved taxonomy of *Geotria* stems from the problems posed by the fact that, unlike Northern Hemisphere lampreys, this genus undergoes radical morphological changes during the protracted spawning run [29, 32, 33]. Adult *Geotria* are characterized by possessing a pair of longitudinal blue-green stripes along the dorsal region of the body, a supraoral lamina with four cusps (the inner smaller and sharply pointed, the outer larger with rounded edges), a transverse lingual lamina with two or three large cusps, two enlarged darkly pigmented oral papillae and a large gular pouch in mature males [24, 25, 29, 33, 34]. During sexual maturation, the blue-green coloration fades to a dull brown, the body length reduces by approximately one third, and the dorsal fins change shape and become closer. In addition, the labial teeth on the oral disc become smaller and widely spaced, the transverse lingual lamina changes from tridentate to bidentate, the relative size of the prebranchial region and the length and width of the oral disc increases, a large gular pouch develops in males [10, 27, 29] and the females develop a ‘rope’, a raised ridge in front of the first dorsal fin [6].

Morphological characters have traditionally been used to identify lamprey species [15, 35, 36]. Although *G*. *australis* undergoes pronounced ontogenetic changes during its freshwater spawning run, Riva-Rossi et al. [4] identified two morphological characters that distinguished Patagonian *G*. *macrostoma* from *G*. *australis* at the immature adult stage. Namely, the second dorsal and caudal fins are connected by a low skin fold and are contiguous in *G*. *macrostoma*, whereas there is a distinct space between the posterior end of the second dorsal fin and the origin of the caudal fin in *G*. *australis*. The gap between the second dorsal fin and the caudal fin in *G*. *australis* has disappeared by sexual maturity, but currently this has only been documented for females in Australia [32, 37]. However, this may not be a gender specific difference and could reflect the paucity of records of mature spawning *G*. *australis*. In addition, the position of the cloaca is always posterior to the origin of the second dorsal fin in *G*. *macrostoma*, whereas it is anterior to, or under, the origin of the second dorsal fin in *G*. *australis*, as is also the case with ammocoetes [13].

Detailed morphological investigations beyond obvious macroscopic descriptors are important, however, for defining and characterizing populations, understanding biodiversity and evolution of the species, as well as supporting ecological knowledge and conservation [36, 38, 39]. The objective of this study on *Geotria* species, was, therefore, to quantify morphometric characters, such as the lengths of various body parts, and physical characteristics, which are traditionally used by lamprey taxonomists to differentiate lamprey species [15]. These characters are then used to determine the features that distinguish adults of *G*. *macrostoma* and *G*. *australis* and thereby further characterize the re-assigned species of Geotriidae.

## Materials and methods

This study was carried out in accordance to the ethical regulations of CONICET (Consejo Nacional de Investigaciones Científicas y Tecnológicas) for biomedical and biological research with laboratory and farm animals and those obtained in nature (Resolution D 1047 Annex II of the year 2005), and in accordance with the NIWA Animal Ethics Committee approval AEC189. Lamprey capture and handling procedures were approved by specific permits issued by the Ministerio de Agricultura, Ganaderia y Pesca from the Río Negro Province (Resolution 007), by the Instituto Provincial del Agua, Administración General de Recursos Hídricos from the Chubut Province (Resolution 24/19DGAguas-IPA), by the Ministerio de Producción, Comercio e Industria, Subsecretaría de Coordinación Pesquera from the Santa Cruz Province (Resolution MPCI 438818/18 del Provincia de Santa Cruz), from the Ministry for Primary Industries, Fisheries New Zealand, (Special Permit 666/2), and from the South Australian Minister for Primary Industries and Regional Development (Section 115 Ministerial Exemption ME9903055). Lamprey anesthesia in Argentina was performed using a mild dose (30 mg/mL) and for mature adult lamprey only, an overdose (100 mg/mL) of benzocaine (Parafarm, CABA, Argentina). For genetic analyses presented in Riva-Rossi et al. [4], a sample of 25 individuals (15 from the Santa Cruz River and 10 from the Chubut River) were euthanized with an overdose of benzocaine, stored at –20˚C, and transported to the laboratory in Puerto Madryn. In New Zealand and Australia lamprey were anaesthetized with 0.05 ml/L stock solution of AQUI-S (AQUI-S, Lower Hutt, New Zealand). As *G*. *australis* is considered a threatened species [40] all individuals were released alive back to the river of capture.

Although Clemens [5] used adult to encompass non-feeding, pre-spawning lamprey that have commenced their upstream migration and are in various stages of sexual maturation, sexual maturation in adult *Geotria* takes around 16 months. Therefore, to clarify the life stages examined in the present study, adult *Geotria* are termed immature when collected on entry to fresh water and show no external sexual dimorphism. Adults that show sexual dimorphism but have not fully matured (i.e. are not ready to spawn) are termed immature male or female. Clemens [5] terms post-spawning lamprey in the process of dying senescent. However, post-spawning *Geotria* remain active for months, exhibiting parental care and guarding of their eggs, therefore, lamprey that show sexual dimorphism at or post-spawning are termed mature adults.

### Sample collection

**Fig 1:**
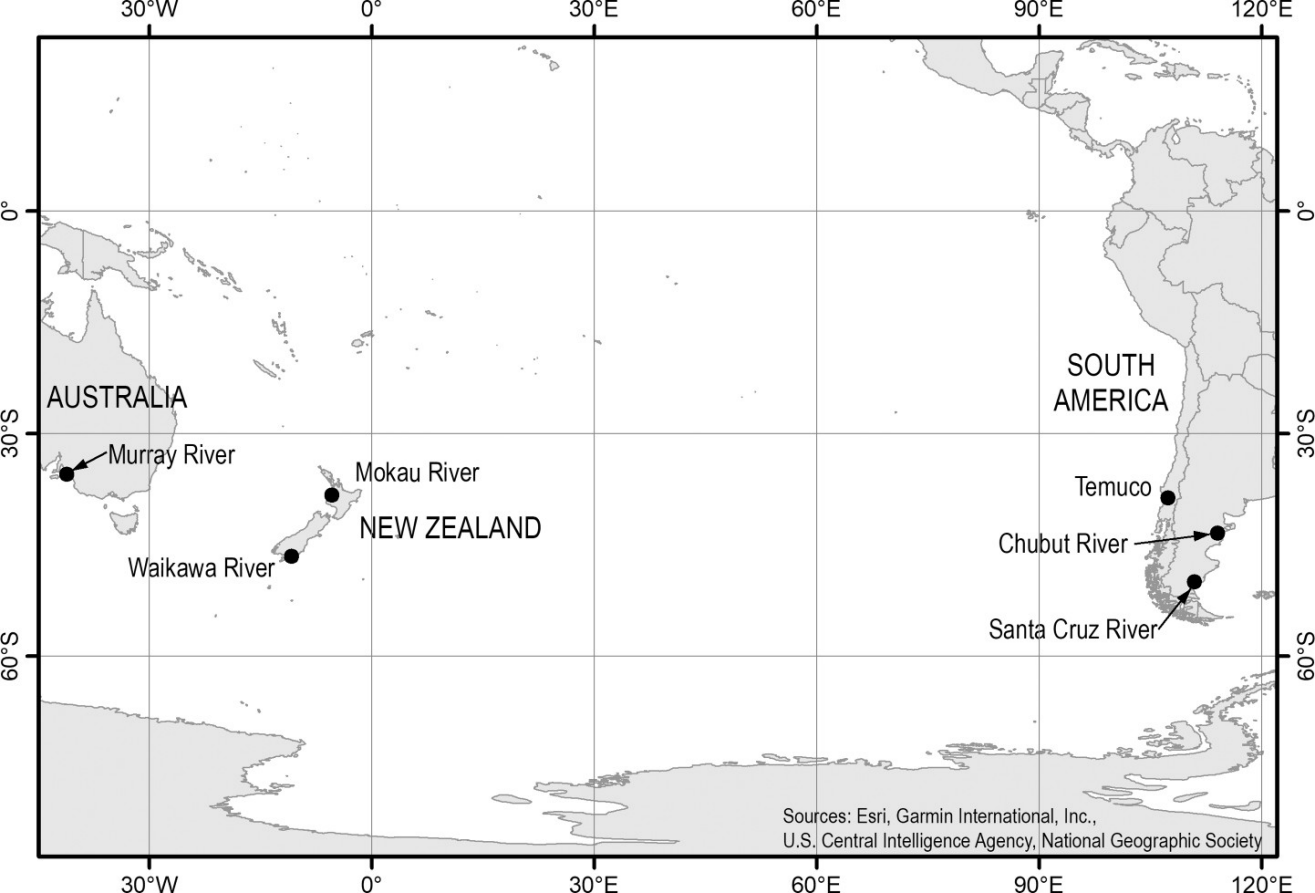
Location of sampling sites in Australia (Murray River), New Zealand (Mokau and Waikawa rivers), Argentina (Chubut and Santa Cruz rivers) and Chile (Temuco). Fig 1 was created using ArcGIS 10.6 based on our own collection sites, geographic information and shapefiles available at NIWA and geographic resources freely available online: https://www.arcgis.com/home/item.html?id=170b5e6529064b8d9275168687880359.

A total of 164 immature adult *G*. *macrostoma* were collected from two sampling sites in Argentina during their upstream migration (Fig 1). Between February and March 2019, 125 *G*. *macrostoma* were captured using fyke nets in the lower Santa Cruz River, Argentina (50.05°S, 69.01°W). In May 2019, 39 *G*. *macrostoma* were collected by electrofishing and by hand from the lower Chubut River, Argentina (43.45°S, 65.94°W). In Australasia, 155 immature adult *G*. *australis* were collected from three sampling sites during their upstream migration (Fig 1). In August 2019, 84 *G*. *australis* were captured by hand from two rock weirs located immediately above the tidal zone in the Waikawa River, Southland, New Zealand (46.59°S 169.14°E). In September 2019 and August 2020, 42 *G*. *australis* were captured using cage traps in fishways on the Murray Barrages in the lower Murray River, Australia (35.53°S, 138.81°E). In August 2020, 29 *G*. *australis* were captured by hand from a rock weir located approximately 84 km inland in the Mokau River, New Zealand (38.32°S 174.58°E). While no fresh run immature adults of *G*. *australis* from Chile were collected as part of this study, eight immature adult *G*. *australis* collected from Temuco, Chile, in October 1963 and measured by Neira [10] were included in the dataset (Fig 1).

As *G*. *australis* undergoes a wide range of morphometric changes during development it was important to examine lampreys from each location at a known point of their freshwater spawning run. To measure fresh run immature adult lamprey upon entry to fresh water, whilst still displaying the blue-green coloration, sampling locations for the Waikawa River, Chubut River, Santa Cruz River and Murray River were in the lower reaches, at or just above tidal influence. The Waikawa River, Chubut River and Murray River individuals were examined within four days of capture during their autumn through spring migration run. The Santa Cruz River lamprey enters fresh water during summer and logistical challenges prevented measuring the entire sample immediately. Twenty-five individuals were measured immediately and released back to the river with the remaining 100 held alive at the Piedra Buena hatchery, Argentina, in a flow through outdoor tank fed directly from the Santa Cruz River. These lamprey were measured six weeks after capture. It is important to note that although the month of river entry varies between the Waikawa, Santa Cruz, Chubut and Murray populations, the lamprey are all entering fresh water in the same state as fresh run immature adults, this is verified by the blue coloration, which they retain during their entire oceanic phase but lose soon after entering fresh water [32]. To determine if time in fresh water and corresponding morphometric growth led to the characters of *G*. *australis* and *G*. *macrostoma* overlapping, lampreys from the Mokau River, New Zealand, were also examined. Migratory *G*. *australis* had already lost their blue-green coloration prior to reaching the inland fishing location in the Mokau River. Based on observations of Baker et al. [6], this indicated that the lamprey had been in fresh water for at least four weeks. Mokau River lamprey were held in the NIWA laboratory for four additional weeks before measurements were taken, which was when early development of the gular pouch became evident. In addition, the lamprey collected from Temuco, Chile, had already lost their blue-green coloration prior to reaching the inland fishing location and, therefore, are comparable to the Mokau River lamprey, having been in fresh water for at least four weeks.

For comparison with the immature adults, measurements from sexually mature adults of both species were included in the dataset. Three post-spawning *G*. *macrostoma* were analyzed. One male was captured in a fyke net set in the lower Santa Cruz River during January 2020 (and released back to the river), while the other specimens had been collected during April 2016 from both the lower Santa Cruz River (male) and during October 2017 from the upper Santa Cruz River (female). The latter two specimens were fixed in 10% neutral buffered formalin and deposited in the Ichthyology Collection of the Instituto de Diversidad y Evolución Austral (IDEAus-CONICET), Puerto Madryn, Argentina. Four sexually mature post-spawning *G*. *australis* from Neira [10] were also included. These lamprey consisted of a male from the Chillán River (captured February 1974), a male from the Maullín River (captured February 1979), a female from the Andalién River (captured May 1981) and another female from Concepción Bay, Talcahuano (captured February 1977). In addition, photographs of a post-spawning male and female *G*. *australis* from Canterbury, New Zealand, have been included in figures for descriptive comparisons with mature *G*. *macrostoma*.

### Morphometric and physical characters

**Fig 2:**
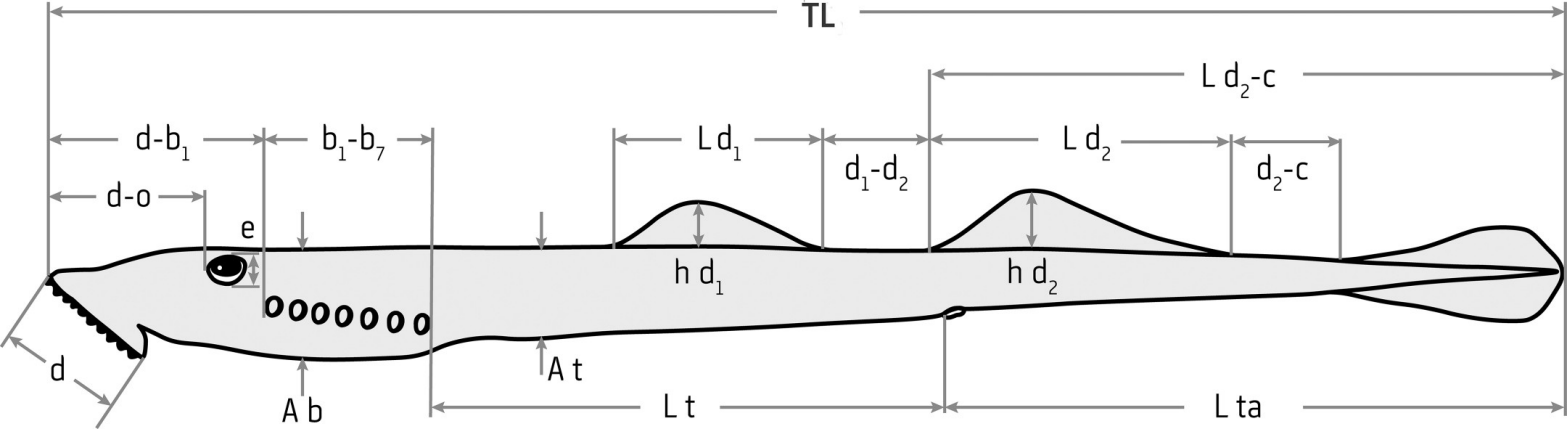
Lateral view of an adult lamprey depicting morphometric measurements. d: oral disc length; d-o: snout to eye length; d–b_1_: prebranchial length; b_1_‑b_7_: branchial length; e: eye height; Ab: maximum branchial depth; At: maximum trunk depth; Lt: trunk length; Lta: tail length; Ld_1_: length of first dorsal fin; hd_1_: height of first dorsal fin; d_1_-d_2_: space between dorsal fins; Ld_2_: length of second dorsal fin; hd_2_: height of second dorsal fin; d_2_-c: space between the posterior end of the second dorsal fin and origin of caudal fin; Ld_2_-c: length from origin of the second dorsal fin to the tip of the caudal fin; TL: total length. Not shown is oral disc width (w).

In total, 22 morphometric characters were measured or calculated, of which, 18 were measured to the nearest 0.01 mm using an electronic caliper (Fig 2). Measurements largely followed Renaud [15] and Neira [10], but eye height as opposed to eye length was measured and the length of the cloacal slit was included in tail length. In addition to the 17 measurements labelled in Fig 2, following Potter et al. [32], the width of the oral disc was measured after it had been splayed out on a glass plate. Precloacal length (pa) was calculated by adding trunk length (Lt) with prebranchial length (d–b_1_) and branchial length (b_1_–b_7_). The oral disc of all lampreys measured was photographed to examine its dentition. Two further morphometric ratios were calculated, oral disc length as a percentage of prebranchial length (d/ d–b_1_ x 100), and tail length as a percentage of the length from the origin of the second dorsal fin to the tip of the caudal fin (Lta/ Ld_2_-c x 100). Gender was not assigned as sexual dimorphism is not apparent externally upon entry to fresh water and most individuals were released alive. In accordance with Renaud [15], morphometrics were taken on the left side of the lamprey (head pointing left) and were measured as the shortest distance point to point (Fig 2).

The number of oral fimbriae of 20 *G*. *australis* from the Waikawa River and 29 *G*. *macrostoma* from the Santa Cruz River were counted from scaled digital images using Image J software (https://imagej.nih.gov/ij/download.html). The total length of 10 oral fimbriae from each of the 49 specimens was measured; five from the antero-lateral region and five from the posterior region of the oral disc. As fimbriae were not excised from the specimens as by Lethbridge & Potter [41], their length was measured from the base of the exposed fimbriae to the tip of the longest finger-like processes. The fimbriae lengths of each lamprey were expressed as a percentage of its oral disc width.

### Condition factor

The condition factor of *G*. *australis* and *G*. *macrostoma* was calculated by using Fulton’s (K) index [42]. For this, the weight of each lamprey was measured to the nearest 1 g and condition factor was calculated using the formula W/TL^3^ x 10^6^, where W is wet weight in g and TL is total length in mm.

### Data analysis

As lamprey length varies between individuals, the various morphometrics were standardised by expressing them as a percentage of total length; except with eye height, fin height and length, and branchial and trunk depth. Note that the absence of a space between the posterior end of the second dorsal fin and origin of the caudal fin (d_2_-c) of *G*. *macrostoma* meant this metric was not available for inclusion in analyses.

All morphometrics were log_10_ transformed and subjected a priori to the Shapiro-Wilks and Levene’s tests to determine if they met the assumptions of normality and homogeneity of variance, respectively. One-way Analysis of Variance’s (ANOVAs) were performed on log_10_ transformed data for each of the remaining 22 morphometric characters from the five lamprey populations (Waikawa River, Mokau River, Murray River, Chubut River and Santa Cruz River; α ≤ 0.05). When significant differences occurred for a given character, Tukey’s Honest Significant Difference (HSD) test was used to determine pairwise differences among lamprey populations. Oral fimbriae were subjected to factorial ANOVAs to identify whether fimbriae size relative to location on the disc differed between the two *Geotria* species. A t-test was carried out to determine if the total number of oral fimbriae was significantly different between the two species.

Standard Discriminant Function Analysis (DFA) was used to determine the morphometrics that could best discriminate between lamprey populations [43]. DFA creates a predictive model for assigning groups, determining which morphometric measurements were the best predictors of lamprey populations based on the percentage of correctly classified individuals. Pearson Product Moment Correlations were first performed to identify co-linearity in the morphometrics measured. Highly correlated variables (>0.85) were removed and only the two variables with a correlation greater than 0.75 were used in the DFA analyses; snout to eye % TL and oral disc length % prebranchial length (0.81), as their inclusion strengthened the discriminant model. The DFA analysis was carried out on log_10_ transformed data from the five immature lamprey populations (Waikawa River, Mokau River, Murray River, Chubut River and Santa Cruz River), the mature adult lamprey from the Santa Cruz River and the immature and mature adult lamprey from Chile [10]. Wilks’s λ was used to compare the differences among groups, which ranges from 0 (perfect discrimination) to 1.0 (lack of discrimination). The eigenvalues, percentage of variance, and cumulative percentage of variance were calculated in this analysis. Discriminant functions or canonical roots were considered useful for explaining the data if the eigenvalues were greater than 1. The standardized coefficients of the canonical roots were determined for estimating the relative contribution of each variable to each of the roots, thus, indicating the power of discrimination for each of the selected variables. Finally, the matrix of structure factors was calculated to determine the intra-group absolute correlations between each of the variables and the canonical root. The larger standardized coefficients and the larger correlations between each variable and the root were utilized to explain the data.

For initial analyses the immature Santa Cruz lamprey were separated into two groups to determine if any morphometric factor changed during the six-week holding period. As no discrimination between the fresh run and older groups was evident, the data were pooled for subsequent analyses.

Morphometric measurements taken from 14 lamprey specimens held at the Muséum National d’Histoire Naturelle (Paris) (measured in May-June 2003) and at the Natural History Museum (London) (measured January 2010), including types and non-types, were also used in descriptive analyses to determine if they exhibit the morphometric descriptors of *G*. *macrostoma*.

All statistical analyses were carried out using Statistica version 13.4.0.14 (TIBCO Software Inc.)

## Results

### Coloration, fin shape and their relative position

**Fig 3:**
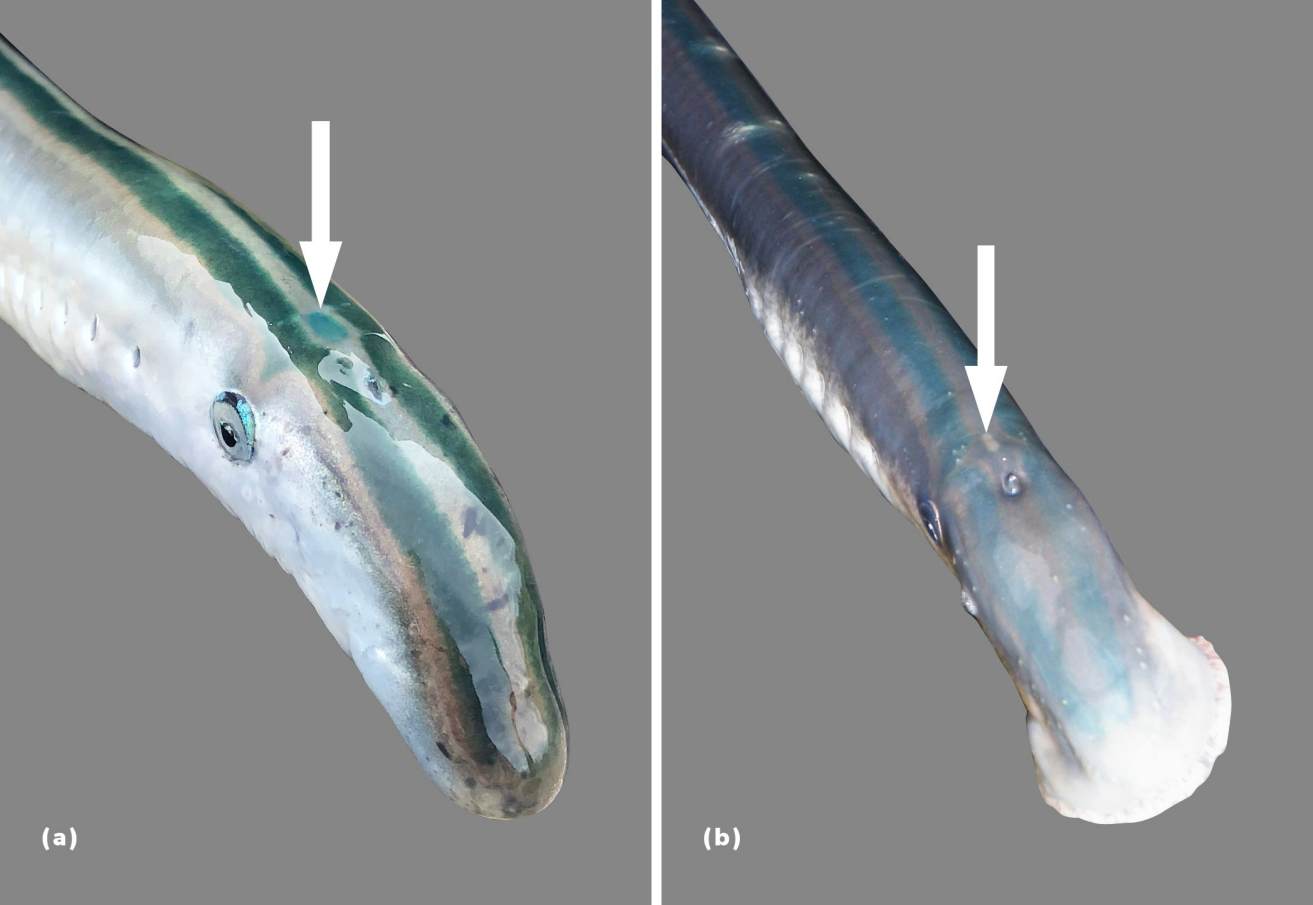
Coloration of fresh run *Geotria* lampreys. a) *G*. *macrostoma* displaying the iridescent blue markings on the outer edge of the eye and over the pineal gland (arrow) and the two blue-green longitudinal stripes characteristic of both *Geotria* species, b) *G*. *australis* lacking iridescent blue markings on the outer edge of the eye and over the pineal gland.

**Fig 4:**
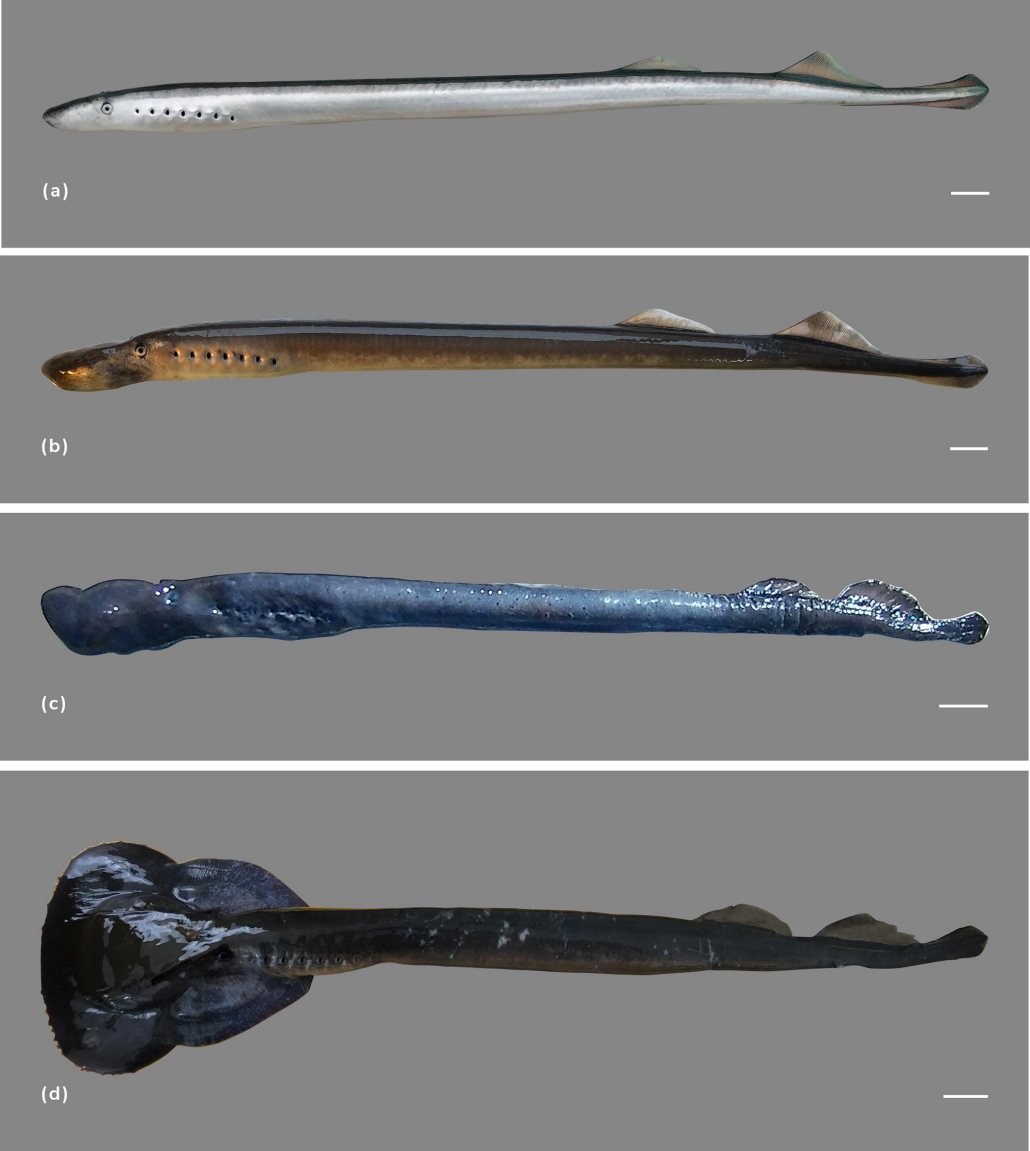
Immature and mature *G*. *macrostoma*. a) fresh run *G*. *macrostoma* displaying the blue-green coloration to the dorsal region, the silver/white ventral surface and the iridescent blue markings along the trailing edges of the dorsal fins and along the entire edge of the caudal fin as well as the fleshy tip of the tail, b) *G*. *macrostoma* after six weeks in fresh water lacking the blue-green coloration to their dorsal region and the loss of iridescent markings to the fin tip, c) sexually mature female *G*. *macrostoma* (assumed post-spawning), d) sexually mature male *G*. *macrostoma* (assumed post-spawning). Scale bars = 2 cm.

As with *G*. *australis*, *G*. *macrostoma* enters fresh water with two dorsal longitudinal blue-green stripes and a blue-green coloration on the upper half of its body, whereas the ventral surface is silver/white (Fig 3). A characteristic of *G*. *macrostoma*, which has not been documented for *G*. *australis*, is the iridescent blue coloration, almost fluorescent in appearance, that is present on the edges of the eyes, over the pineal gland and along the trailing edges of the dorsal fins and entire edge of the caudal fin, as well as on the fleshy tip of the tail (Figs 3 and 4). In contrast, the skin over the pineal gland is creamy/white in *G*. *australis* with no iridescent blue markings on the edges of the eyes and fins. After six weeks in fresh water, the Santa Cruz River lamprey no longer displayed the fin markings and most had lost their vibrant blue-green coloration, fading to dull brown (Fig 4).

**Fig 5:**
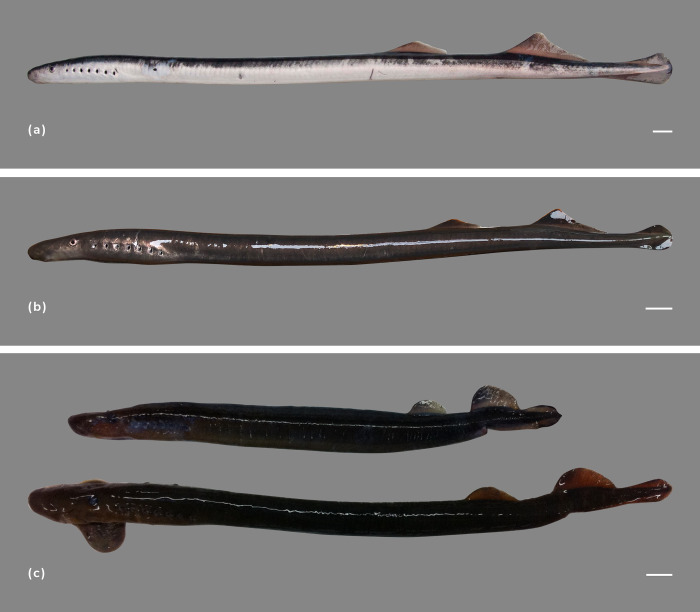
Immature and mature *G*. *australis*. a) fresh run individual, b) after approximately four weeks in fresh water, c) post-spawning female (top) and post-spawning male (bottom). At sexual maturity the second dorsal and caudal fins in both sexes are contiguous. Scale bars = 2 cm.

One of the diagnostic characteristics of immature *G*. *macrostoma* is the contiguous second dorsal and caudal fins (Fig 4; [4]). In Australian *G*. *australis*, the loss of a space between the posterior end of the second dorsal fin and the origin of the caudal fin has only been documented in mature females not males [32, 37]. In sexually mature New Zealand *G*. *australis*, the two fins are contiguous in both sexes (Fig 5). At sexual maturity, the dorsal fins of *G*. *australis* lose their peaked triangular shape and the apex becomes rounded (Fig 5). The rounding of both dorsal fins, and particularly of the second dorsal fin is less pronounced in mature *G*. *macrostoma* (Fig 4). Mature female *G*. *macrostoma* lack the raised dorsal ridge (rope), which develops in front of the first dorsal fin in mature female *G*. *australis* (Fig 5). A large gular pouch is possessed by the mature males of both *Geotria* species (Figs 4 & 5).

### Dentition and oral fimbriae

**Fig 6:**
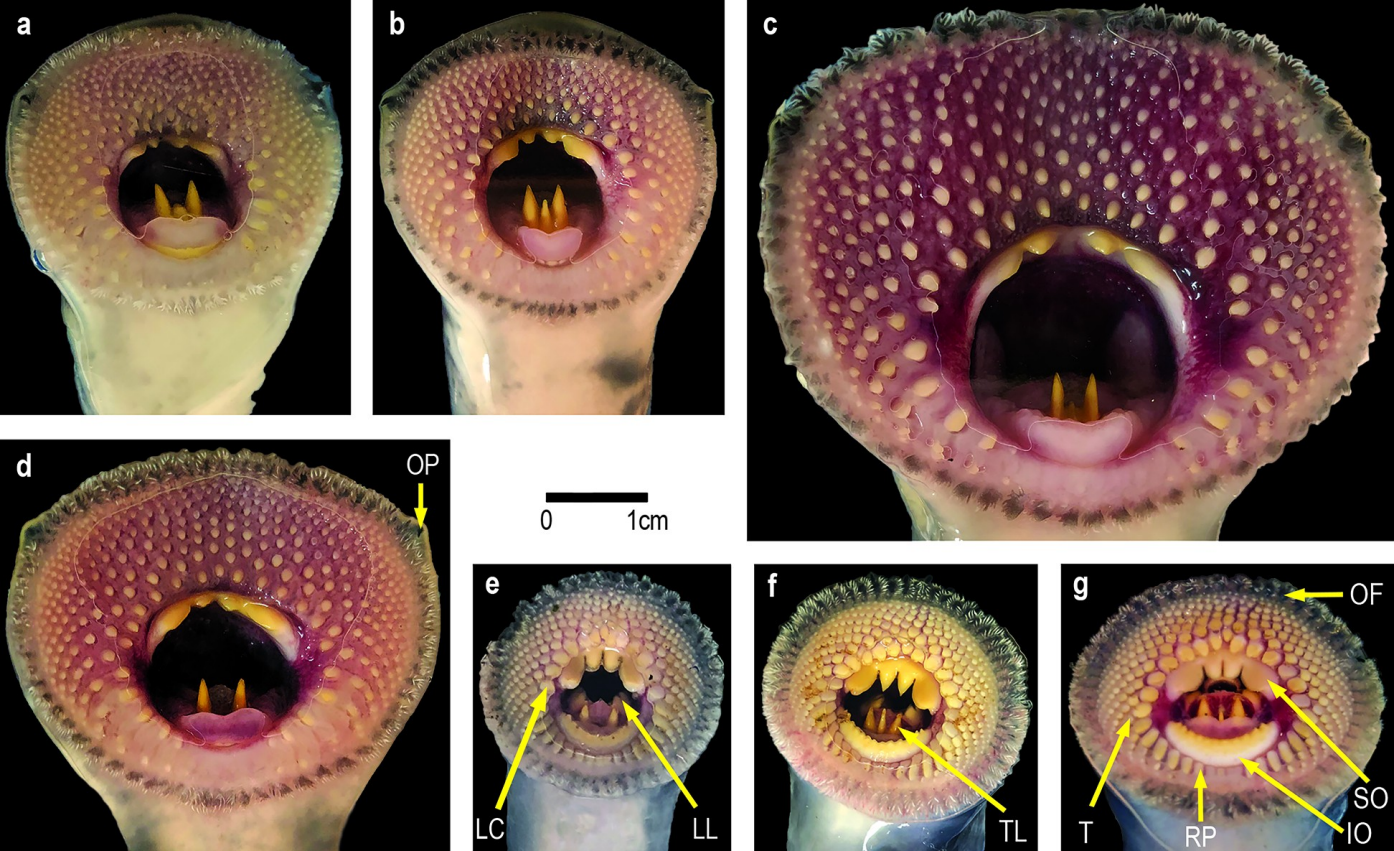
Dentition of immature *G*. *macrostoma* (a-d) and *G*. *australis* (e-g): IO: infraoral lamina; LC: lateral circumoral; LL: longitudinal lingual lamina; OF: oral fimbria; OP: oral papilla; RP: radial plate; SO: supraoral lamina; T: labial teeth; TL: transverse lingual lamina.

In general, the number of cusps on each lamina of *G*. *australis* and *G*. *macrostoma* were the same. Both *Geotria* species possess four cusps on the supraoral lamina, three or two large cusps on the transverse lingual lamina, four or five cusps on both longitudinal lingual laminae, and 8–11 small cusps on the infraoral lamina (Fig 6). There is one enlarged darkly pigmented oral papilla on either side of the oral disc in both species (Fig 6). However, the shape of the teeth and changes undergone between immature and mature *G*. *macrostoma* differ markedly from those of *G*. *australis*.

Fig 6E–6G displays the variation seen in the shape and arrangement of cusps on each lamina in New Zealand *G*. *australis*. The shape and arrangement of teeth agree with those documented in the published literature [27, 32, 44]. In immature *G*. *macrostoma* key differences are: the infraoral lamina is either reduced or absent (Fig 6A–6D) whereas it is retained in *G*. *australis* throughout its spawning run [32]; the two central pointed teeth in the supraoral lamina are markedly smaller with the outer cusps not displaying the characteristic spatulate shape, but instead consist of smaller elongated cusps; the remaining teeth in the oral disc are not spatulate as in *G*. *australis*, particularly the lateral circumorals (adjacent to the oesophageal opening); and the single row of ridge-like radial plates in the posterior section of the disc in *G*. *australis* are reduced in number or absent in *G*. *macrostoma*.

**Fig 7:**
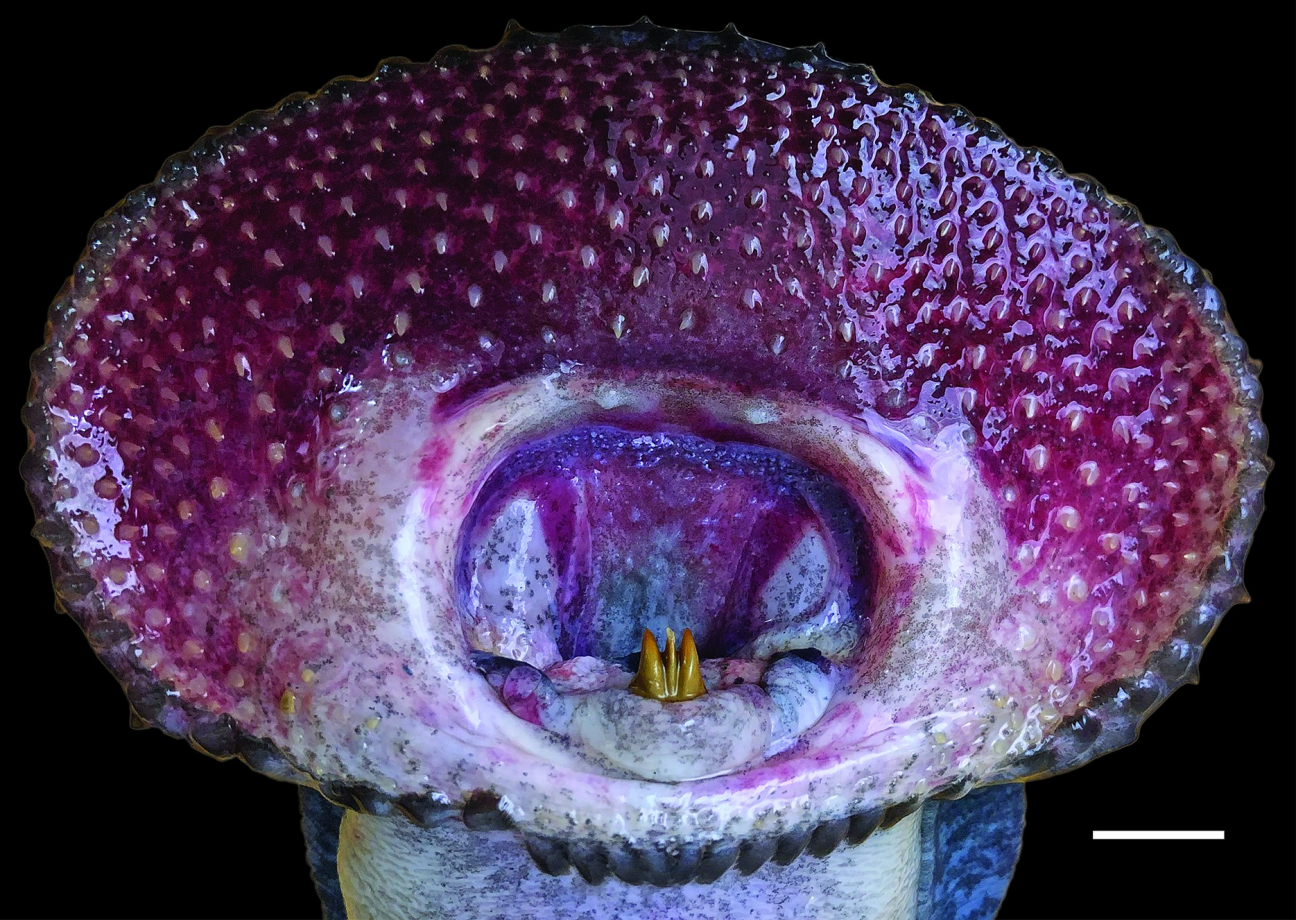
Dentition of a mature male *G*. *macrostoma*. Scale bar = 1 cm. The transverse lingual lamina is tricuspid showing two lateral and one central tooth.

In *G*. *australis*, aside from the two outer cusps of the transverse lingual lamina, the cusps of the other laminae and teeth on the oral disc become smaller and blunter as lamprey reach sexual maturity [32, 44]. This reduction in tooth size is also seen in sexually mature *G*. *macrostoma* (Fig 7). Although the infraoral lamina disappears early in the spawning run, the supraoral lamina has become markedly reduced to two small triangular cusps by sexual maturity (Fig 7). In addition, the growth of the oral disc is unprecedented for any lamprey species with a width of over 100 mm in mature *G*. *macrostoma*; more than double the documented disc width for *G*. *australis*.

**Fig 8:**
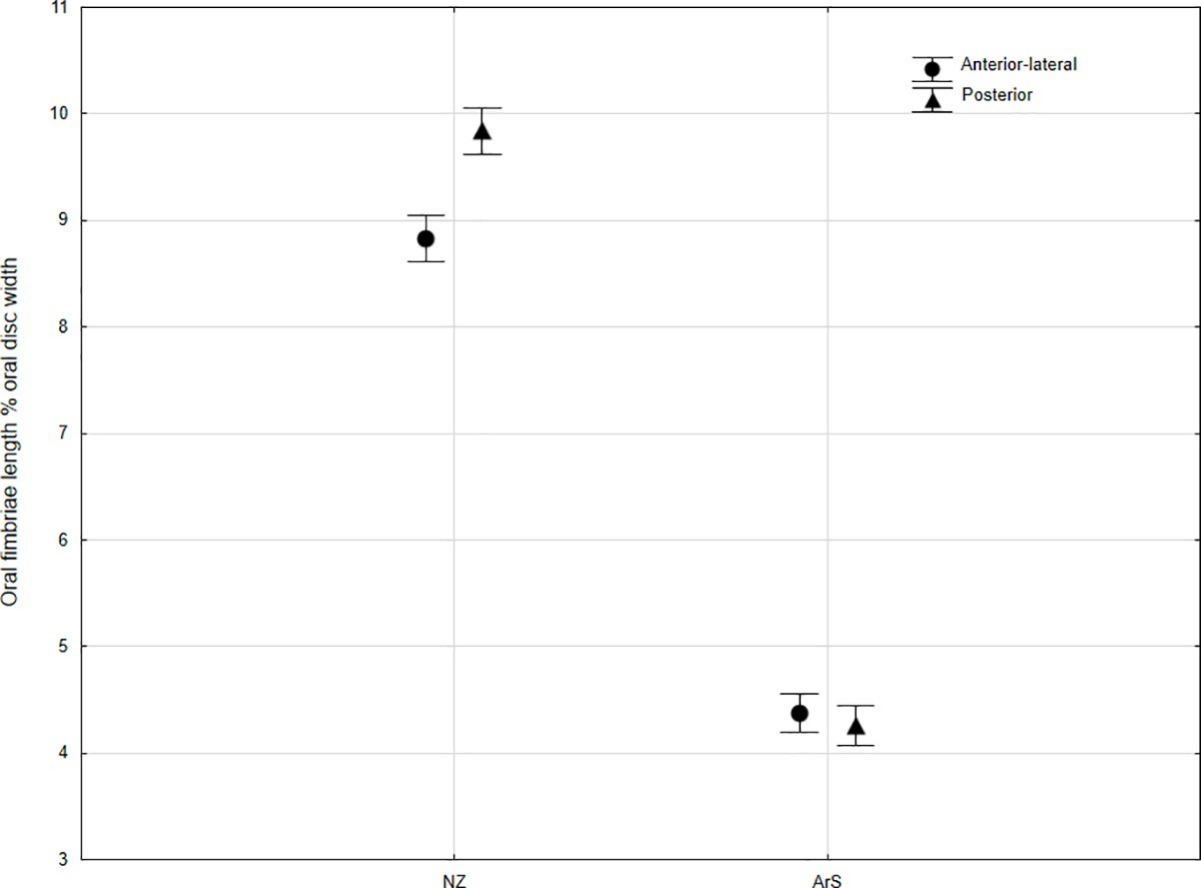
Plot of oral fimbriae length as a percentage of oral disc width for immature NZ *G*. *australis* from the Waikawa River and immature *G*. *macrostoma* from the Santa Cruz River. For both anterior-lateral and posterior regions, the mean ± 95% confidence interval is displayed. Error bars that do not overlap depict significant differences between regions and lamprey species (*p*<0.05).

Another difference between the species is the number and size of the oral fimbriae. *G*. *macrostoma* possessed between 67 and 76 oral fimbriae, which was significantly more than the 53 to 64 recorded in *G*. *australis* (*P <* 0.0001; Table 1). The length of the oral fimbriae as a percentage of the oral disc width was also significantly smaller in *G*. *macrostoma* than in *G*. *australis* (*P <* 0.0001; Fig 8). For *G*. *macrostoma*, no significant difference was found in the size of oral fimbriae between the anterior-lateral and posterior regions (Fig 8). In contrast, the anterior-lateral oral fimbriae were significantly smaller than those from the posterior region of *G*. *australis* (*P <* 0.0001; Fig 8). Although only three mature adult *G*. *macrostoma* could be examined, erosion/loss of the finger-like processes was evident in all individuals with the oral fimbriae presenting as fleshy nodes (c.f. Figs 6 & 7). In contrast, mature adult *G*. *australis* retain their oral fimbriae finger-like processes throughout adult life [6, 32].

**Table 1:** Comparisons of morphological characters of immature and mature adults of *Geotria australis* and its synonyms with those recorded in the present study of six lamprey populations (Chubut and Santa Cruz rivers, Argentina; Temuco, Chile; Waikawa and Mokau rivers, New Zealand; Murray River, Australia), three mature *G*. *macrostoma* (Santa Cruz River, Argentina) and four mature *G*. *australis* (Chillán, Andalién and Maullín rivers and Concepción Bay, Chile).

Taxon	Catalogue Number	Locality	Stage	Total Length (mm)	Oral disc length (mm)	Oral fimbriae count	Oral papillae count	Oral disc length % TL	prebranch % TL	branch % TL	trunk % TL	tail % TL	precloacal % TL	oral disc length % prebranch length	snout to eye % TL
*Geotria australis*, non-type P	MNHN 1989–1205	Argentina: lower Negro River	IF	431.0	28	71	23	6.5	14.15	9.74	53.36	22.04	77.26	45.90	10.44
*Geotria australis*, non-type P	MNHN 1989–1205	Argentina: lower Negro River	IM	440.0	36	67	24	8.2	15.80	10.00	54.66	20.45	80.45	51.80	8.18
*Geotria australis*, non-type	BMNH 1936.6.12.1	Chile: Tumbes, Talcahuano	IF	313.0	19	ND	ND	6.1	13.58	9.74	61.50	15.18	84.82	44.71	11.02
*Velasia chilensis*, holotype	BMNH 1951.10.4.2	Chile	I	376.5+	16	50	17	4.2	11.16	10.49	59.10	17.40	80.74	38.10	7.04
*Geotria australis*, non-type P	MNHN A–8056	Australia: Yarra River drainage	I	478.5	13	ND	ND	2.7	8.78	9.20	59.98	22.88	77.95	30.95	6.69
*Geotria australis*, non-type L	BMNH 1964.4.30.3	New Zealand: Hutt River, North Island	I	397.0	25	60	17	6.3	15.11	9.70	55.42	17.88	80.23	41.67	10.58
*Geotria saccifera*, holotype P	BMNH 1886.11.18.112	New Zealand: Otago, South Island	IM	420.0	25.5	58	17	6.1	15.36	10.36	57.38	16.90	83.10	39.53	10.48
*Geotria australis*, non-type P	BMNH 1935.3.14.1	New Zealand: Island Bay, North Island	IF	483.0	17.5	59	16	3.6	11.49	9.94	59.42	18.94	80.85	31.53	6.83
*Geotria australis*, non-type	MNHN 714	Chile	MM	363.0	25	ND	ND	6.9	16.25	10.88	54.82	18.18	81.96	42.37	11.02
*Geotria allporti*, holotype	BMNH 1871.8.18.51	Australia: Hobart, Tasmania	MM	342.0	30	ND	ND	8.8	19.30	11.11	49.27	21.49	79.68	45.45	13.60
*Geotria australis*, non-type R	BMNH 1908.12.28.97	Australia: South Australia	MM	386.0	32	50	19	8.3	18.13	10.23	54.92	16.84	83.29	45.71	12.69
*Geotria australis*, non-type	BMNH 1911.4.1.72	Australia: Tasmania	MM	473.5	38.5	ND	ND	8.1	16.79	11.09	53.75	17.63	81.63	48.43	12.35
*Geotria australis*, holotype	BMNH 1851.7.11.1	Australia: Inkar pinki River	MM	487.0	41.5	59	17	8.5	17.45	10.57	55.85	15.09	83.88	48.82	12.94
*Geotria australis*, non-type	MNHN A–8058	New Zealand	MM	426.0	22	ND	ND	5.2	12.68	10.33	56.10	18.43	79.11	40.74	8.10
**This study**															
*Geotria macrostoma*		Santa Cruz River, Argentina	MM	470.0	51.0	ND	ND	10.9	20.2	9.9	52.1	17.2	82.2	53.7	16.3
*Geotria macrostoma*		Santa Cruz River, Argentina	MF	412.0	40.1	ND	ND	9.7	17.9	9.8	54.1	17.7	81.8	54.3	17.3
*Geotria macrostoma*		Santa Cruz River, Argentina	MM	438.0	70.4	ND	ND	16.1	23.7	10.7	48.9	17.4	83.3	67.7	19.5
*Geotria australis*		Estero Las Piedras, Chile	MM	490.0	38.0	ND	ND	7.8	14.2	10.4	55.5	23.4	76.5	54.6	10.0
*Geotria australis*		Maullín River, Chile	MM	435.0	37.7	ND	ND	8.7	15.6	9.8	59.7	16.1	83.9	55.5	12.5
*Geotria australis*		Andalien River, Chile	MF	405.0	21.5	ND	ND	5.3	11.9	8.9	55.8	21.2	79.0	44.7	9.4
*Geotria australis*		Concepción Bay, Talcahuano, Chile	MF	270.0	20.9	ND	ND	7.8	20.2	11.8	47.6	16.3	76.8	38.4	15.0
*Geotria macrostoma*		Chubut River, Argentina	I	580 (532–629)	30.8 (23.3–39.2)	ND	ND	5.3 (4.0–7.0)	12.0 (10.2–14.6)	9.7 (9.0–10.7)	57.9 (52.5–61.3)	21.2 (19.4–23.8)	79.6 (73.3–82.9)	44.1 (38.4–53.9)	8.5 (6.8–10.6)
*Geotria macrostoma*		Santa Cruz River, Argentina	I	508 (429–569)	32.0 (22.1–46.2)	67–76	ND	6.3 (4.4–9.0)	12.9 (10.2–16.4)	9.8 (8.2–12.1)	56.5 (52.6–60.0)	21.4 (18.5–23.6)	79.2 (75.3–82.5)	48.5 (37.7–56.5)	9.4 (7.0–12.6)
*Geotria australis*		Waikawa River, New Zealand	I	544 (438–623)	19.8 (16.7–23.7)	53–64	ND	3.6 (3.1–4.5)	9.7 (8.9–10.8)	9.4 (8.4–10.5)	57.9 (52.8–60.2)	23.3 21.2–25.2)	77.0 71.3–79.6)	37.4 (32.3–43.0)	6.3 (5.5–7.3)
*Geotria australis*		Mokau River, New Zealand	I	574 (533–609)	22.0 (19.4–26.2)	ND	ND	3.8 (3.3–4.7)	9.8 (8.9–10.8)	9.8 (8.9–10.7)	57.7 (54.2–60.9)	23.4 (21.4–25.9)	77.3 (74.5–79.6)	39.2 (35.6–43.7)	6.3 (5.4–7.2)
*Geotria australis*		Murray River, Australia	I	557 (530–604)	20.9 (18.8–24.9)	ND	ND	3.7 (3.5–4.2)	9.5 (9.3–9.8)	9.9 (9.3–10.6)	58.5 (57.0–60.0)	23.0 (21.5–24.4)	77.9 (76.1–79.5)	39.3 (36.6–43.9)	6.1 (5.7–6.6)
*Geotria australis*		Temuco, Chile	I	504 (445–570)	13.8 (12.5–16.1)	ND	ND	2.8 (2.3–3.1)	9.1 (8.3–9.9)	8.9 (8.1–10.0)	56.5 (51.0–61.5)	25.2 (21.1–31.1)	75.3 (68.4–79.8)	30.1 (27.1–33.9)	6.8 (5.7–7.6)

Abbreviations: MNHN: Muséum National d’Histoire Naturelle, Paris; BMNH: National History Museum, London; TL: total length; I: immature, undetermined gender; IF: immature female; IM: immature male; MM: mature male; MF: mature female; P: specimen with prominent ridges of epithelium; L: specimen with low ridges of epithelium; R: specimen with remnants of epithelial ridges; prebranch: prebranchial region; branch: branchial region; ND: not determined. For the five lamprey populations in the present study and the immature lamprey from Chile [10], the mean value for each morphometric is provided with the range given in brackets. + signifies a damaged caudal fin so total length could not accurately be measured. Hence caution is needed in interpreting all measures standardised by length.

### Morphometrics and condition

**Fig 9:**
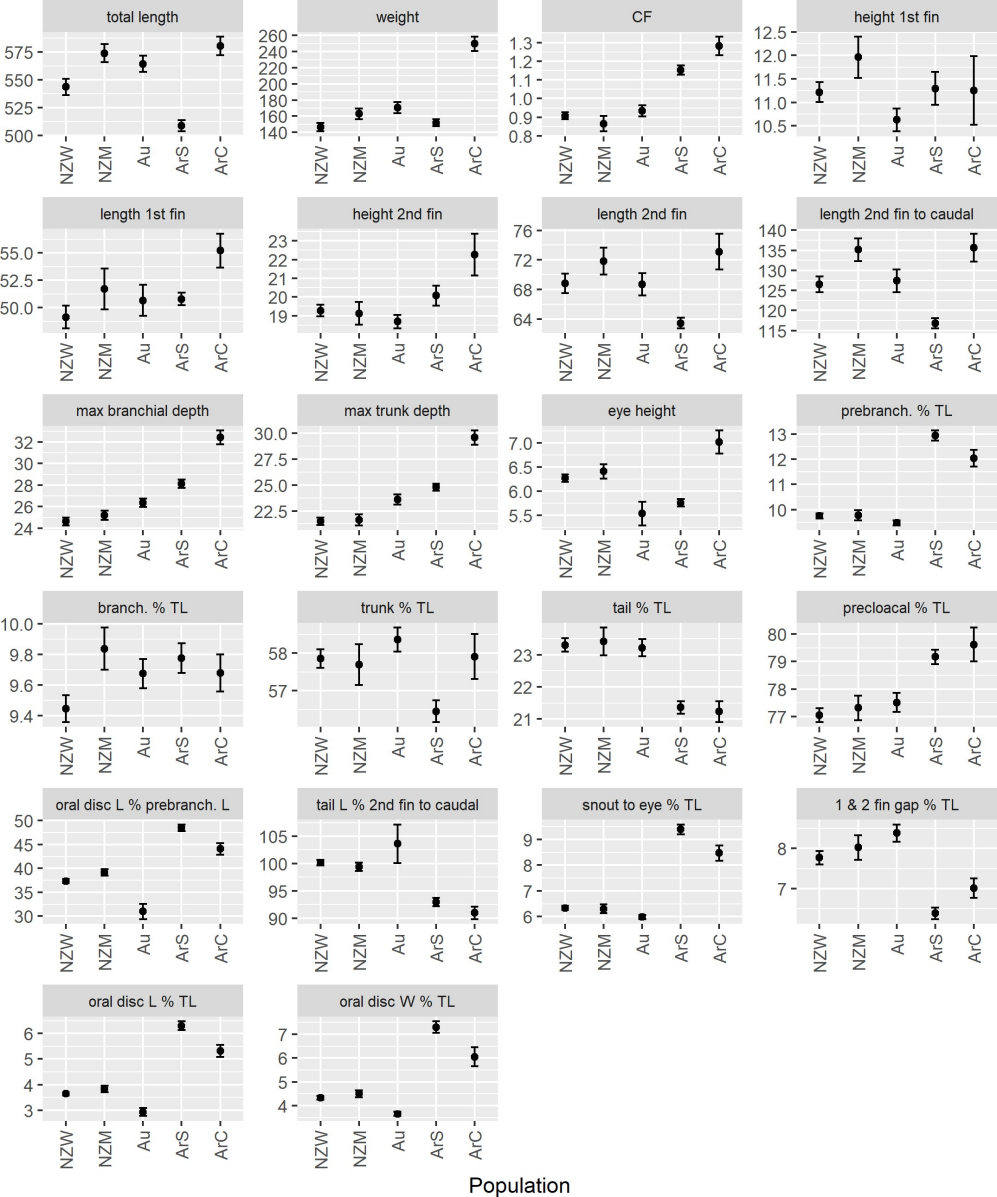
Plots of the 21 morphometric characters and condition factor. For each character, the mean ± 95% confidence interval is displayed. All characters were significantly different among the five lamprey populations (NZW: Waikawa River, New Zealand; NZM: Mokau River, New Zealand; Au: Murray River, Australia; ArS: Santa Cruz River, Argentina; ArC: Chubut River, Argentina). Error bars that do not overlap depict significant differences between lamprey populations (*P* < 0.05). Abbreviations: TL: total length; CF: condition factor; W: width. All measurements are in millimetres and weight is measured in grams. Note d_2_-c (space between the posterior end of the second dorsal fin and origin of the caudal fin) is not graphed as it is absent in *G*. *macrostoma*.

The ANOVA’s found all 21 morphometric variables and condition factor were significantly different among the five lamprey populations (Fig 9). A number of characters differed significantly between the two species at the time of their entry to fresh water. The prebranchial region and oral disc of *G*. *macrostoma* was larger (Fig 9), resulting in the prebranchial and snout to eye lengths, and length and width of the oral disc being significantly greater than in all *G*. *australis* populations (*P <* 0.05; Fig 9). As the cloaca of *G*. *macrostoma* is positioned further behind the origin of the second dorsal fin than in *G*. *australis* [4], *G*. *macrostoma* has a significantly smaller tail length, and tail length forms a significantly smaller proportion of the region from the origin of the second dorsal fin to the end of the caudal fin (*P <* 0.05; Fig 9). The precloacal region, branchial depth and trunk depth of *G*. *macrostoma* were also significantly larger than that of *G*. *australis* (*P <* 0.05). Differences between the species are also reflected in the total length, weight and condition factor (Fig 9). *G*. *macrostoma* was heavier relative to total length and exhibited a significantly higher condition factor than *G*. *australis* (*P <* 0.05).

The branchial and trunk lengths, eye height, and the height and length of the two dorsal fins did not show a clear pattern between the two *Geotria* species (Fig 9). However, the space between the two dorsal fins was significantly greater in *G*. *australis* than *G*. *macrostoma* (*P <* 0.05; Fig 9).

Within species differences were also evident, with *G*. *macrostoma* populations more heterogeneous than *G*. *australis*. *G*. *macrostoma* from the Chubut River exhibited significantly larger branchial and trunk depths and space between the two dorsal fins than those from the Santa Cruz River (*P <* 0.05; Fig 9). *G*. *macrostoma* from the Chubut River was also significantly longer and heavier, and with a significantly higher condition factor than lamprey from the Santa Cruz River (*P <* 0.05). *G*. *macrostoma* from the Santa Cruz River exhibited significantly larger oral disc length and width, snout to eye and prebranchial regions from those in the Chubut River (*P <* 0.05). Although the gular pouch had started to develop in Mokau River lamprey, differences in the morphometrics measured were minimal, with only the branchial region, total length and weight being significantly larger than Waikawa River lamprey (*P <* 0.05; Fig 9). Australian *G*. *australis* also exhibited larger trunk depths and a smaller sized oral disc compared to New Zealand populations (*P <* 0.05).

**Fig 10:**
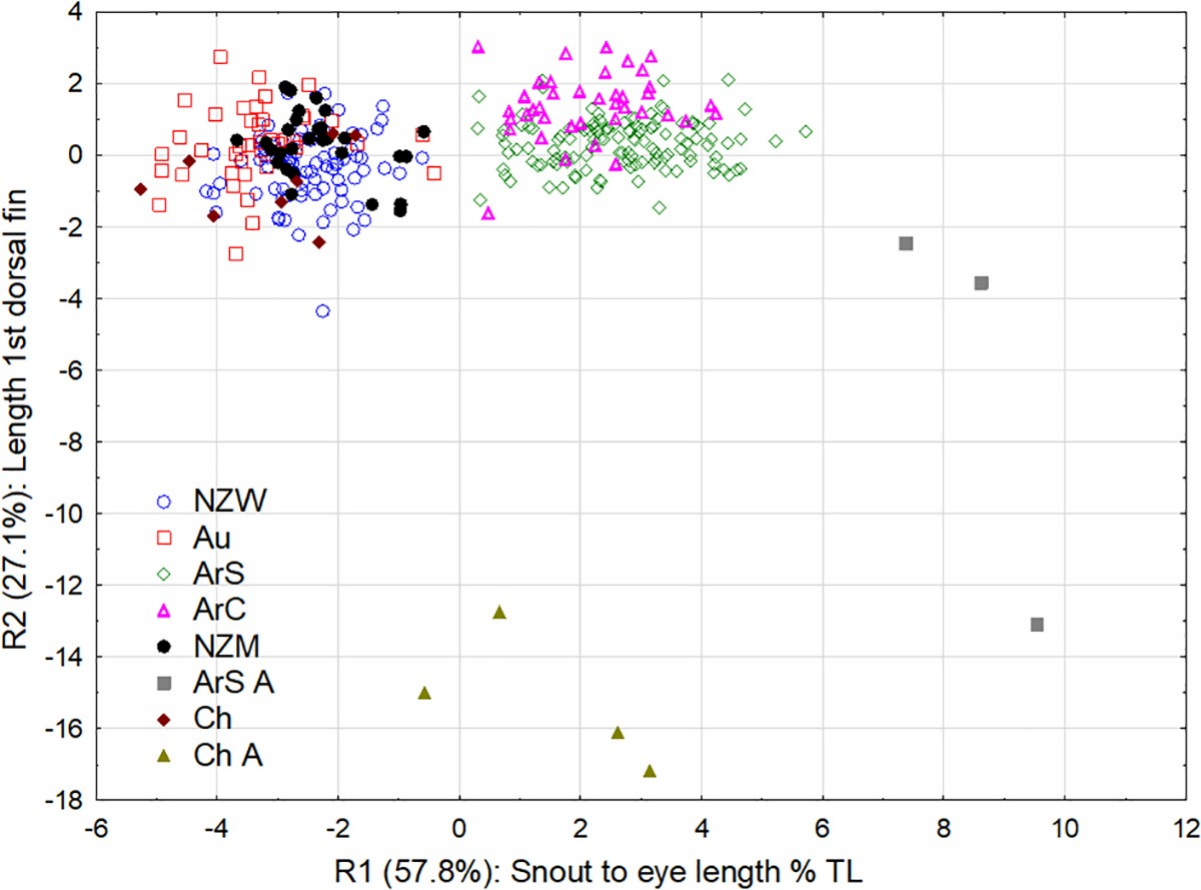
Factor plot showing canonical scores of population means for the first two discriminant functions (canonical roots). Root 1 (R1) was positive so the higher the score the larger the snout to eye length of the lamprey relative to total length, and root 2 (R2) was positive, so the higher the score the larger the first dorsal fin length. (NZW: Waikawa River, New Zealand; NZM: Mokau River, New Zealand; Au: Murray River, Australia; ArC: Chubut River, Argentina; ArS: Santa Cruz River, Argentina; Ch: Temuco, Chile; ArS A: mature adult lamprey from the Santa Cruz River, Argentina; Ch A: mature adult lamprey from four Chilean rivers).

The Discriminant Function Analysis (DFA) consisted of eight variables: length of the first dorsal fin, height of the second dorsal fin, maximum trunk depth, oral disc length as a percentage of prebranchial length, and four characters standardised by total length; snout to eye length, tail length, space between the first and second dorsal fins and precloacal length. Of the eight variables in the DFA, all except precloacal length were found to be significant characters (*P <* 0.01). The DFA clearly separated immature *G*. *australis* and *G*. *macrostoma*, and mature adult individuals of *G*. *macrostoma* from the Santa Cruz River were different from all immature populations and mature adult *G*. *australis* from Chile (Wilks’λ = 0.0059, F_56,1712_ = 48.62, *P <*0.00001). Three canonical roots were generated by the DFA with eigenvalues higher than 1. Root 1 had an eigenvalue of 7.65 explaining 57.8% of the total variance, while root 2 had an eigenvalue of 3.59 explaining 27.1% of the total variance and root 3 had an eigenvalue of 1.10 explaining 8.3% of the total variance, accounting for a cumulative proportion of 93% (Fig 10; Table 2).

**Table 2:** Structure matrix and standardised canonical coefficients resulting from the discriminant function analysis on immature and mature *G*. *australis* and *G*. *macrostoma*.

Morphometric	Structure matrix			Standardised canonical coefficients		
	Root 1	Root 2	Root 3	Root 1	Root 2	Root 3
Snout to eye length %TL	**0.807**	-0.241	0.098	**0.655**	0.018	-0.001
Oral disc length % prebranchial length	**0.638**	-0.051	0.416	0.402	0.055	0.466
Length first dorsal fin	-0.032	**0.928**	0.092	0.062	**0.918**	0.194
Max trunk depth	0.316	0.271	**-0.797**	0.435	0.238	**-0.750**
Height second dorsal fin	0.101	-0.139	-0.256	-0.334	-0.313	-0.283
Space between first and second dorsal fins %TL	-0.380	0.137	-0.252	-0.186	0.058	-0.225
Precloacal length % TL	0.267	0.011	-0.179	-0.001	0.179	-0.057
Tail length % TL	-0.354	0.143	0.188	-0.123	0.253	0.270

The structure matrix highlights intra-group correlations between each of the morphometrics and the discriminant functions. Morphometrics with greater contribution in each discriminant function are in bold. The canonical coefficients estimate the relative contribution of each morphometric to the discriminant model. The greatest significant values are in bold.

Of the seven statistically significant variables in the DFA, the largest standardized coefficient in the first canonical root was the snout to eye length as a percentage of total length (0.655), followed by maximum trunk depth (0.435) and oral disc length as a percentage of prebranchial length (0.402; Table 2). Length of the first dorsal fin was the most important variable influencing root two (0.918), followed by height of the second dorsal fin (0.313) and tail length as a percentage of total length (0.253). Maximum trunk depth was the most important variable influencing root three (-0.750) followed by oral disc length as a percentage of prebranchial length (0.466) and height of the second dorsal fin (0.283). Accordingly, the structure matrix showed that the snout to eye length as a percentage of total length and oral disc length as a percentage of prebranchial length were the variables that showed the highest correlation with the first canonical axis, whereas length of the first dorsal fin and maximum trunk depth were the most closely related variables to the second and third axis, respectively (Table 2).

The DFA model discriminated well between lamprey populations with 84.6% of original grouped cases correctly classified (Fig 10; Table 3). Incorrect classifications were only found within species, with no *G*. *macrostoma* classified as *G*. *australis* and vice versa (Table 3). The Mahalanobis distances were significantly different between all lamprey populations (*P <* 0.025). Comparing the six immature populations, Mahalanobis distances also indicated a stronger separation between the *G*. *australis* and *G*. *macrostoma* species (25.4–57.8) than different populations of the same species (1.0–16.5 for New Zealand, Australia and Chile *G*. *australis*, and 7.7 for Santa Cruz and Chubut *G*. *macrostoma*). Mature adults from the Santa Cruz River also displayed a strong separation from mature *G*. *australis* from Chile (195.4).

**Table 3:** Classification matrix from the discriminant function analysis on morphometric characters among the eight lamprey populations (NZW: Waikawa River, New Zealand; NZM: Mokau River, New Zealand; Au: Murray River, Australia; ArC: Chubut River, Argentina; ArS: Santa Cruz River, Argentina; Ch: Temuco, Chile; ArS A: Mature adult lamprey from the Santa Cruz River, Argentina; Ch A: Mature adult lamprey from four Chilean rivers).

	Predicted classifications (%)								
Population	% correct classification	NZW	Au	ArS	ArC	NZM	ArS A	Ch	Ch A
NZW	95.2	95.2	2.4	0	0	2.4	0	0	0
Au	78.0	14.6	78.0	0	0	7.3	0	0	0
ArS	96.8	0	0	96.8	3.2	0	0	0	0
ArC	82.1	0	0	17.9	82.1	0	0	0	0
NZM	10.3	89.7	0	0	0	10.3	0	0	0
ArS A	100	0	0	0	0	0	100	0	0
Ch	87.5	12.5	0	0	0	0	0	87.5	0
Ch A	100	0	0	0	0	0	0	0	100

The overall classification was 84.6% correctly predicted.

**Fig 11:**
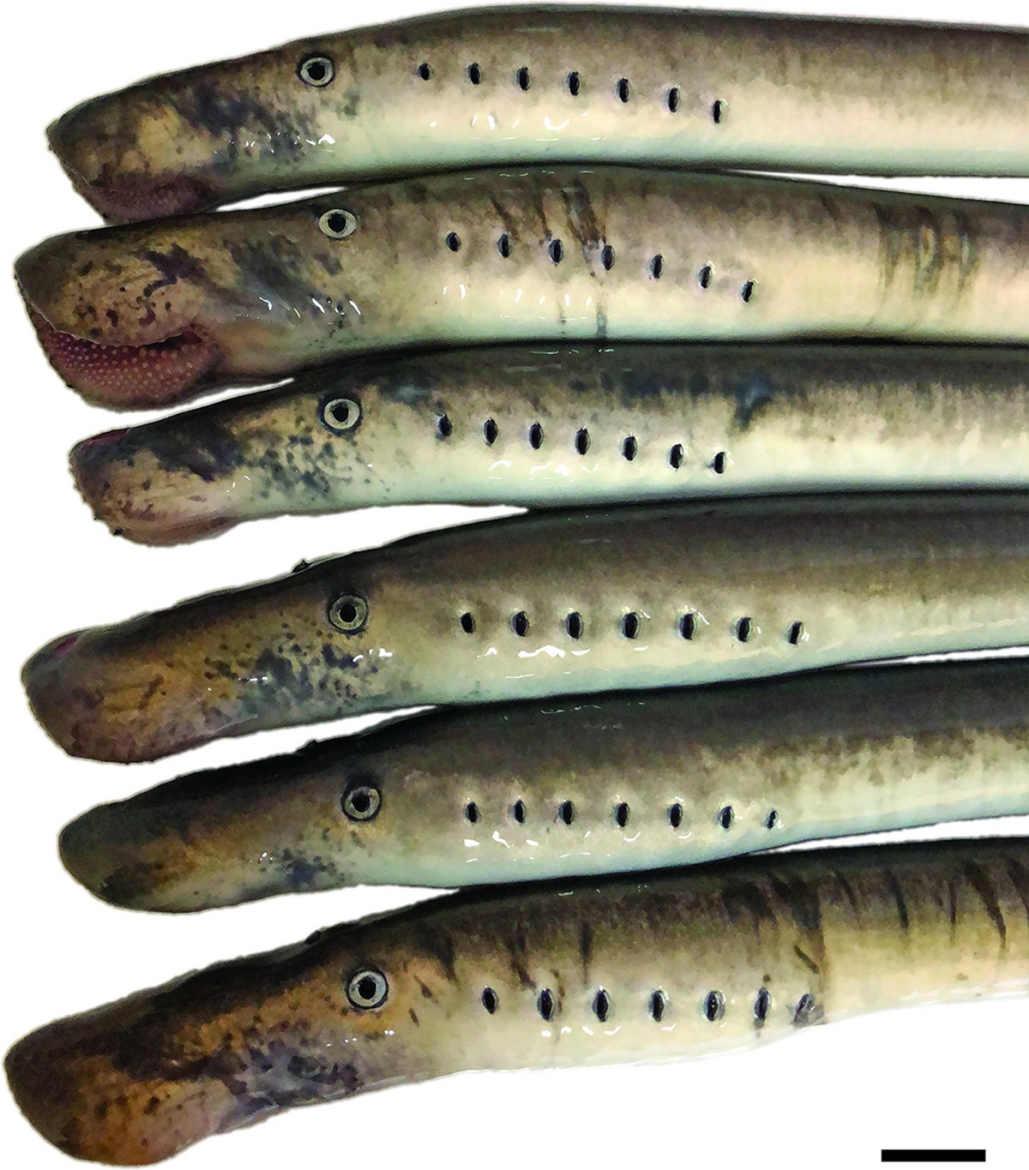
Prebranchial and branchial regions of six *G*. *macrostoma* from the Santa Cruz River showing the variation in size from snout to eye and size of the oral disc. Scale bar = 2 cm.

Overall, DFA results indicated that *G*. *macrostoma* from the Santa Cruz and Chubut rivers had significantly larger snout to eye lengths relative to total length, larger trunk depths and larger oral discs relative to the length of the prebranchial region than *G*. *australis* from New Zealand, Australia and Chile. *G*. *macrostoma* from the Chubut River also had a significantly larger trunk depth, and a longer first dorsal fin than those from the Santa Cruz River (Figs 9 & 10). The difference and variation in size of the snout to eye and oral disc regions in *G*. *macrostoma* is illustrated by Figs 6 and 11. By sexual maturity, *G*. *macrostoma* had significantly larger snout to eye lengths relative to total length than *G*. *australis*. In addition, the length of the first dorsal fin of sexually mature *G*. *macrostoma* and *G*. *australis* was significantly shorter than in immature adults.

The key morphometrics discriminating *G*. *macrostoma* from *G*. *australis* are also highlighted by comparing immature lampreys in the present study with sexually mature *G*. *australis* from Western Australia measured by Potter et al. [32]. As raw data from Potter et al. [32] were unavailable, analyses are limited to descriptive comparisons of means and 95% confidence limits. The prebranchial length of mature male and female *G*. *australis* was, on average, respectively 13.2 and 12.1% of their total length in Australia [32] and 15.5% of their total length (sexes combined) in Chile, whereas the mean prebranchial length of *G*. *macrostoma* (sexes combined) measured 12.0–12.9% of their total length upon entry to fresh water (Table 1). As *G*. *macrostoma* becomes sexually mature, the prebranchial length measures up to 23.7% of their total length (Table 1).

The oral disc of *G*. *macrostoma* also undergoes greater growth through to maturity than that of *G*. *australis*. Upon entry to fresh water, the oral disc length of *G*. *australis* averaged 2.8–3.8% of their total length (Table 1), whereas the oral disc length of *G*. *macrostoma* averaged 5.3–6.3% of their total length (Table 1). Once *G*. *australis* reaches sexual maturity, Potter et al. [32] found the average oral disc length had increased to around 5% of their total length, with Chilean adults reaching 8.7%, whereas the oral disc length of the three mature *G*. *macrostoma* was recorded as 9.7%, 10.9% and 16.1% of their total length (Table 1).

Condition factor was also a significant measure distinguishing *G*. *macrostoma*. Potter et al. [32] found across seven years (1976–1982) the mean condition factor of male and female *G*. *australis* captured during the first four months of their spawning run was between 0.79 and 0.99, respectively. These values are similar to that recorded for *G*. *australis* in the present study (Fig 9). However, the mean condition factor for *G*. *macrostoma* was significantly higher at 1.15 and 1.28 for Santa Cruz and Chubut River lamprey, respectively (Fig 9).

**Fig 12:**
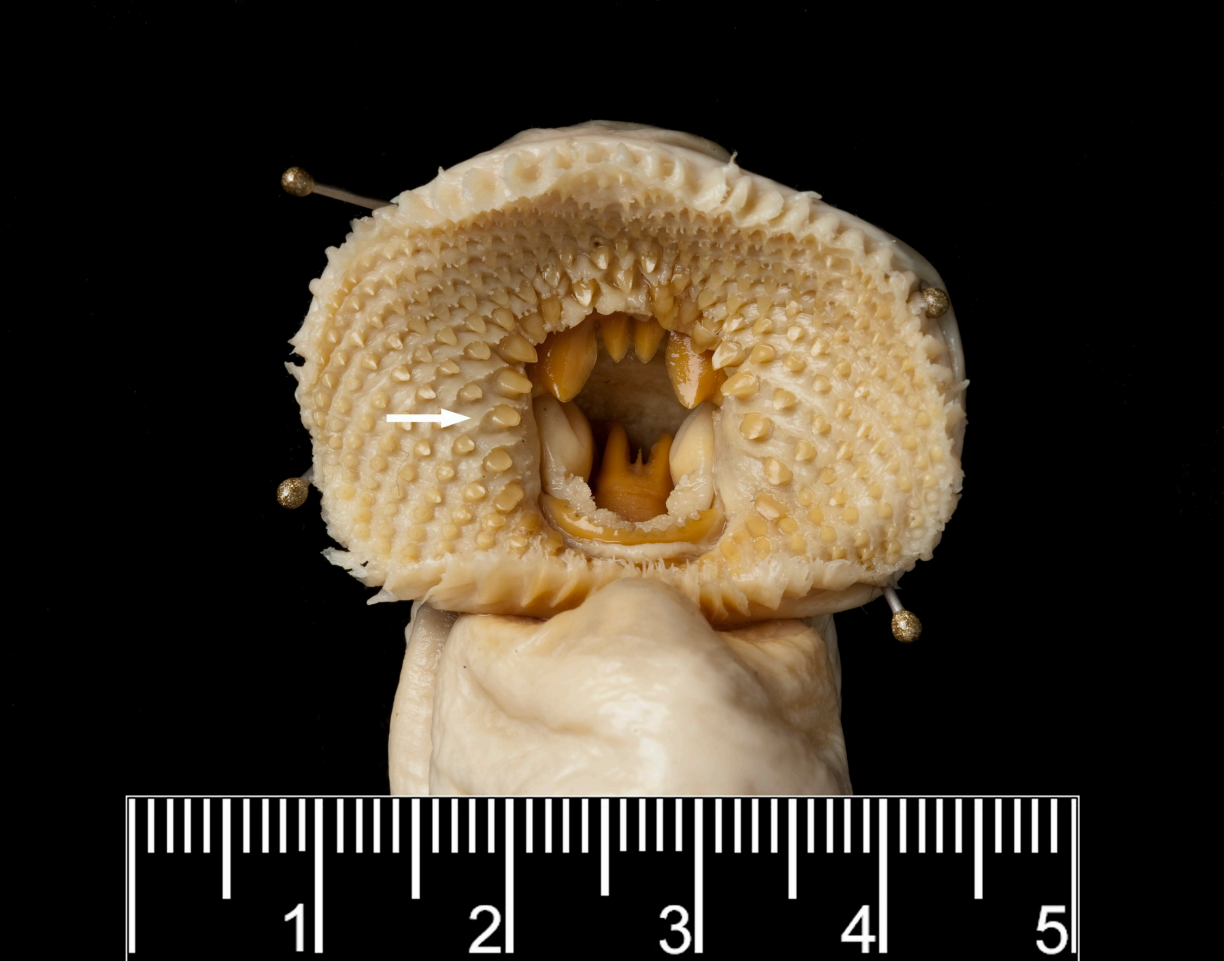
Oral disc of immature adult holotype of *Geotria saccifera* (BMNH 1886.11.18.112) showing the prominent ridges of epithelium flanking the labial teeth (arrow). Photographed by Phil Hurst, Photographic Unit, © The Natural History Museum, London.

**Fig 13:**
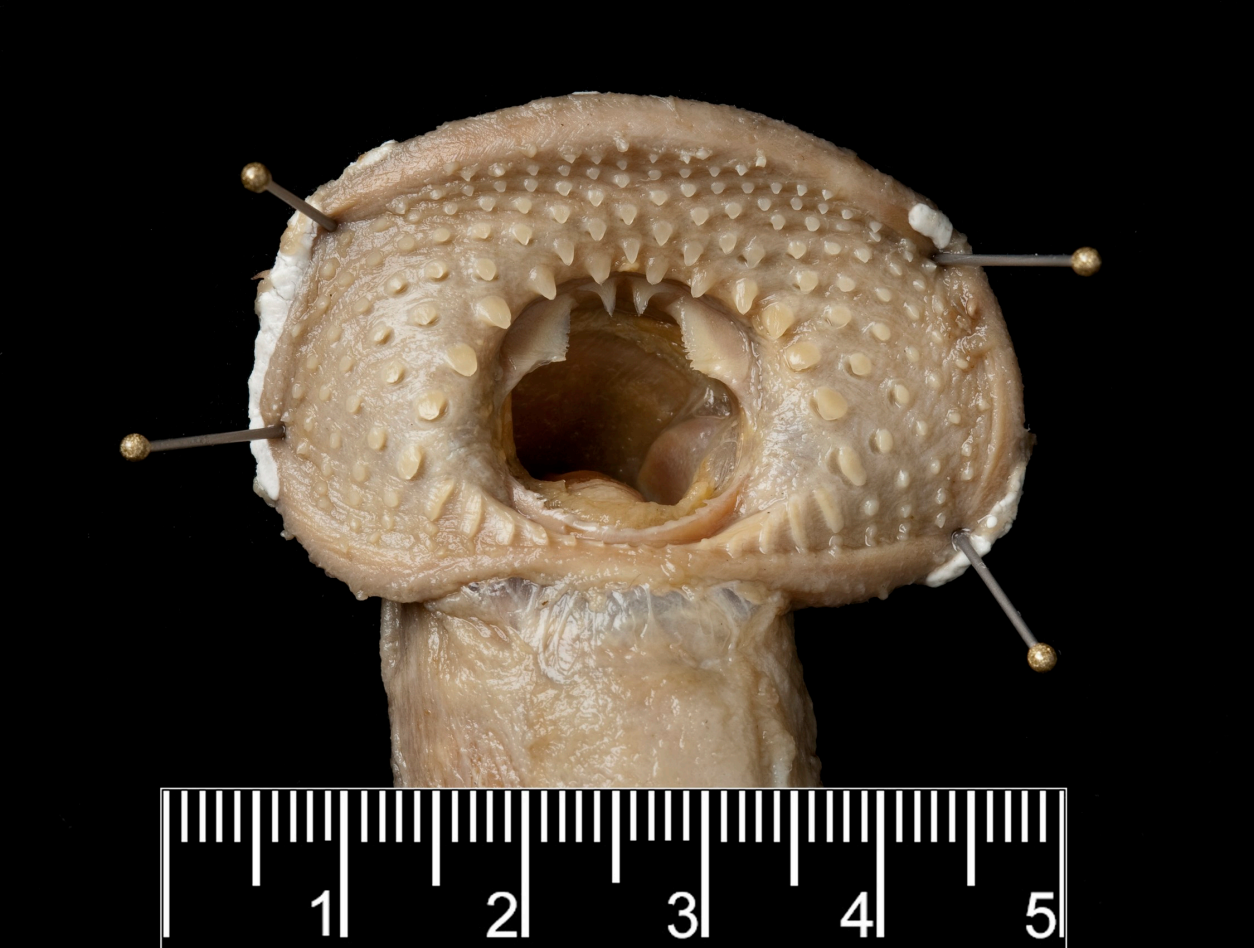
Oral disc of mature adult holotype of *Geotria allporti* (BMNH 1871.8.18.51) without any ridges of epithelium flanking the labial teeth. Note the serration along the inner edge of both lateral flanges of the supraoral lamina reported by Günther [45] in the original description. Photographed by Phil Hurst, Photographic Unit, © The Natural History Museum, London.

A comparison of morphometric characters between immature and mature *G*. *macrostoma* and museum specimens of *G*. *australis*, including its holotype, and those of *G*. *allporti* Günther, 1872, *G*. *saccifera* Regan, 1911, and *Velasia chilensis* Gray, 1851 (synonyms of *G*. *australis*) was also undertaken (Table 1). Although specimens are labelled velasia (immature) or adult, this was based on external characteristics and the exact timing since entry to fresh water is unknown. Six of the eight immature individuals possessed ridges of epithelium flanking the labial teeth (Fig 12) and in five of the six mature adults these were absent (Fig 13); with the other mature adult still showing remnants (Table 1). Of the eight specimens classified as immature, in seven individuals the prebranchial length as a percentage of total length exceeded that recorded in fresh run New Zealand, Australian and Chilean *G*. *australis*, fitting within the range in *G*. *macrostoma* (Table 1). Similarly, for the snout to eye length as a percentage of total length, five of eight specimens exceed that recorded in fresh run *G*. *australis* in the present study (Table 1). At the immature stage, the tail length as percentage of total length of *G*. *australis* should be greater than in *G*. *macrostoma* based on the cloaca being located further forward. However, four of the eight immature *G*. *australis* specimens had relative tail lengths smaller than that measured in any of the six lamprey populations (Table 1). Therefore, it is likely that the variability recorded in the morphological characters across the museum specimens demonstrates the growth of the oral disc, prebranchial and snout to eye lengths in *G*. *australis* paired with the shrinking of total length during the protracted spawning run (Table 1). However, the key morphometrics (oral disc length, and prebranchial and snout to eye lengths as percentages of total length) of the two *G*. *australis* specimens from the Negro River, Argentina, fell within the range of those seen in *G*. *macrostoma* in the present study and outside of the range recorded for *G*. *australis* (Table 1). In particular, the oral disc length (absolute and as a percentage of total length and prebranchial length) were larger than in all other immature *G*. *australis* museum specimens.

The number of oral fimbriae and papillae were also diagnostic in differentiating the specimens. In the two individuals from the lower Negro River, Argentina, the fimbriae and papillae counts were 67–71 and 23–24, respectively (Table 1). In comparison, the six *G*. *australis* that included the holotypes of *G*. *australis*, *G*. *saccifera*, *Velasia chilensis*, and non-type adults (North Island, New Zealand and South Australia, Australia), the fimbriae and papillae counts were 50–60 and 16–19, respectively (Table 1). The range of oral fimbriae in the two lower Negro River specimens matches that seen in immature *G*. *macrostoma* from the Santa Cruz River (Table 1).

A comparison of the three sexually mature *G*. *macrostoma* with mature museum specimens highlights the extensive growth of the key morphometrics (oral disc length, and prebranchial and snout to eye lengths) through the spawning run, relative to that seen in *G*. *australis* (Table 1). Of the six mature museum specimens, oral disc absolute length and prebranchial length as a percentage of total length only overlapped with the values in one and two mature *G*. *macrostoma* specimens, respectively (Table 1). For the snout to eye length as a percentage of total length and oral disc length as a percentage of total length, no mature *G*. *australis* specimen exhibited values as high as those seen in mature *G*. *macrostoma*.

## Discussion

This study has highlighted a range of morphometric and physical characteristics that discriminate between *Geotria* in Patagonian rivers and those found in Australasia and Chile (Table 4). According to Renaud [15], the taxonomy of lampreys is based primarily on the dentition in the adult stage, and the present study identified several characteristics that can be used to discriminate the two *Geotria* species during the immature adult stage (Table 4). In *G*. *macrostoma*, the infraoral lamina is greatly reduced or absent, the supraoral lamina does not display the spatulated outer cusps, the remaining teeth of the oral disc are pointed rather than spatulated (particularly the lateral circumorals), and the posterior ridge-like radial plates present in *G*. *australis* are absent. *G*. *macrostoma* also exhibits an iridescent blue/green coloration to the edges of the eyes, fins and over the pineal gland; however, this coloration fades soon after entry to fresh water. Although not diagnostic, *G*. *macrostoma* displays greater growth of the prebranchial region and oral disc and has a deeper body depth and higher condition factor.

**Table 4:** Diagnostic characters in immature adults of *Geotria*.

Character	*G*. *australis*	*G*. *macrostoma*	Source
Second dorsal and caudal fins	separate	contiguous	Riva-Rossi et al. (2020)
Position of cloaca	under the origin of or anterior to the second dorsal fin	posterior to the origin of the second dorsal fin	Riva-Rossi et al. (2020)
Iridescent blue color on edges of eyes, over pineal gland, along trailing edges of dorsal fins and entire edge of caudal fin, and fleshy tip of tail	absent	present	Present study
Oral fimbriae	50–68	67–76	Present study
Oral papillae	15–22	23–24	Present study
Size of anterior and posterior oral fimbriae	posterior ones larger	same size	Present study
Outer cusps of supraoral lamina	spatulate	shallow and elongate	Present study
Lateral circumorals and labial teeth	spatulate	small	Present study
Infraoral lamina	prominent	reduced or absent	Present study
Row of ridge-like radial plates in the posterior field	present	reduced in number or absent	Present study

The results of this study add to the molecular and morphological differences documented by Riva-Rossi et al. [4] between Patagonian lamprey and those from Chile and Australasia, to validate that *G*. *macrostoma* is a distinct species. There are numerous diagnostic characters that distinguish the two *Geotria* species as they enter fresh water as immature adults; the position of the cloaca and the contiguous second dorsal and caudal fins (described by Riva-Rossi et al. [4]), and four related to the dentition and coloration (found in the present study). The fact that the cloaca of adult *G*. *macrostoma* is located posterior to, rather than under the origin or in front of the second dorsal fin as in *G*. *australis*, parallels the difference found between ammocoetes of *Geotria* from Argentina and those from Chile, Australia and New Zealand [13]. The ammocoetes measured by Neira et al. [13] from the Limay River in Argentina most likely represented *G*. *macrostoma* and those from the other regions *G*. *australis*. Therefore, the difference in the cloaca position in ammocoetes will distinguish between the two *Geotria* species at the larval stage, for which there are fewer well-defined morphological distinctions among lamprey species compared to adults.

The oral fimbriae and papillae were also diagnostic characteristics between *G*. *australis* and *G*. *macrostoma*. Although both species exhibited a large gap devoid of oral papillae along the posterior aspect of the oral disc perimeter, the number of papillae in *G*. *macrostoma* (23–24) exceeded that of *G*. *australis* in the present study (16–19) and those previously reported for Australasia. In seven *G*. *australis* from Australia, Khidir and Renaud [46] reported counts of 16–19 oral papillae, while Maskell [27] reported a range of 15–22, usually 18, for the species in New Zealand. *G*. *macrostoma* also possessed higher numbers of oral fimbriae than *G*. *australis*. The counts of 67–76 recorded in the present study are similar to the 72–74 reported for the holotype of *G*. *macrostoma* by Burmeister [17]. The lower number of oral fimbriae recorded in New Zealand *G*. *australis* in the present study (53–64) fit within the range documented for Australian populations, where Khidir and Renaud [46] reported counts of 55–65 and Lethbridge and Potter [41] reported counts of 50–68. The size of the oral fimbriae relative to location on the disc also differed between the *Geotria* species. In line with the findings of Lethbridge and Potter [41] for Australian *G*. *australis*, the fimbriae in the posterior of the disc in New Zealand *G*. *australis* were significantly larger than those in the anterior and lateral regions. In contrast, *G*. *macrostoma* did not exhibit detectable differences in fimbriae size between disc regions.

Collectively, the present data and investigations by Nardi et al. [16] and Riva-Rossi et al. [4] support the resurrection of *G*. *macrostoma* as a distinct species found in Argentina, with historical records of only two individuals from Uruguay [19] and one adult individual in the San Juan River, a coastal stream flowing into the Chilean side of the Strait of Magellan, on the extreme southern tip of South America [30]. Based on a sole specimen, the presence of *G*. *macrostoma* in southern Chile is questionable. Further assessments of ammocoetes from rivers flowing into the Magellan Channel and/or the Pacific Ocean are warranted to confirm its presence in Chilean Patagonia. Presently, data indicate that *G*. *macrostoma* is likely to be an endemic species to the Patagonian region of Argentine including their South Atlantic islands.

*Petromyzon macrostomus* was first described by Burmeister [17] with Berg [19] re-assigning the species as *Geotria macrostoma* (Burmeister, 1868), adding to the description with a second specimen collected near the island of Flores, off Montevideo, Uruguay. The original description was based on a single specimen of 400 mm total length collected on 26 Sept. 1867 from a street in Buenos Aires. The oral disc is 60 mm in length (oral disc length as percentage of total length, 15) and 80 mm in width. The oral fimbriae number 72–74. A 40 mm long gular pouch reaches the first branchial opening. The snout to eye length is 70 mm (snout to eye length as percentage of total length, 17.5). There are two triangular-shaped dorsal fins separated from each other by 20 mm. The cloaca is under the anterior part of the second dorsal fin and 70 mm from the tip of the caudal fin (tail length as percentage of total length, 17.5). The Patagonian lamprey in this study (Chubut and Santa Cruz rivers) fits with the original description of *Geotria macrostoma*, thereby confirming the former’s identity. Examination of historical descriptions and material suggest museum specimens from the Negro River, Argentina should also be re-identified as *G*. *macrostoma*.

The marked morphological changes that occur in *Geotria* during the protracted spawning run has led to a longstanding unresolved taxonomy of the genus. Comparison of morphometric measures between adult *G*. *macrostoma* and *G*. *australis* revealed that both *Geotria* species are characterized by significant morphological changes through the spawning run with the growth of the oral disc and snout to eye lengths (relative to total length) of sexually mature *G*. *macrostoma* exceeding that seen in *G*. *australis* and all other lamprey species [15, 32]. Morphological differences both among and within lamprey species have been linked to differences in behavior, which may affect ecological processes. For example, Pacific lamprey with a shorter distance between the first and second dorsal fins (that were closer to sexual maturity) were more likely to use refuges during passage at Bonneville Dam [47] and had lower passage success in an experimental vertical slot fishway [48]. The present study has identified marked differences between the two *Geotria* species, especially regarding growth of the oral disc. In particular, in sexually mature *G*. *macrostoma* the finger-like processes of the oral fimbriae are lost/eroded whereas *G*. *australis* retain the full structure throughout its adult life [6, 32]. Lethbridge and Potter [41] suggested that the fimbriae are linked to creating an effective seal on surfaces, not only for feeding and migration but also during nest construction and spawning. As the teeth of *G*. *australis* reduce in size and sharpness during maturation, Lethbridge and Potter [41] speculated that lamprey may become more dependent upon the fimbriae to aid attachment during reproduction. Although conjecture, the loss/erosion of the oral fimbriae finger-like processes and the excessive growth of the oral disc in mature adult *G*. *macrostoma* could indicate ecological differences between the *Geotria* species during migration and breeding. Further investigations are needed to fully understand how morphological differences between the *Geotria* species affect behavior and ecology.

Within the two Patagonian lamprey populations examined in the present study, differences in characters recorded may be the result of differences in the parasitic oceanic phase. *G*. *macrostoma* collected from the Chubut River were significantly larger and heavier with deeper trunks and less growth of the head region than lamprey in the Santa Cruz River. These differences could relate to the time spent at sea and timing of entry to fresh water. In the Chubut River, adult *G*. *macrostoma* enter the river during fall, similar to that observed in Australasian *G*. *australis* [6, 7, 49]. In contrast, in the Santa Cruz River, *G*. *macrostoma* initiated river entry during summer (December through February), similar to Chilean *G*. *australis* [10] and Northern Hemisphere lamprey species [50, 51]. Further investigations are necessary to understand the migration patterns of *G*. *macrostoma* and *G*. *australis* across South American rivers and how these relate to morphometric variation within each species.

Alternatively, the morphological discrimination between the two *G*. *macrostoma* populations may be the result of population structure within the species. Using morphological characters and heart fatty acid signatures, Lança et al. [52] suggested three separate stocks existed in sea lamprey (*Petromyzon marinus*) populations in Portugal possibly based on seabed topography and geographical separation of oceanic host species off the western Iberian Peninsula. From morphological characteristics, Vatandoust et al. [53] also suggested two independent populations of Caspian lamprey (*Caspiomyzon wagneri*) had formed across two major rivers flowing into the Caspian Sea basin. Based on the findings of the present study, further molecular and morphological investigations of *G*. *macrostoma* from within its range are warranted to verify if population structure is occurring within the species.

## Conclusions

The present study and investigations by Nardi et al. [16] and Riva-Rossi et al. [4] support the resurrection of *G*. *macrostoma* as a distinct species inhabiting the major Patagonian basins. Upon entry to fresh water, key morphometric and physical characteristics that discriminated adult *G*. *macrostoma* from *G*. *australis* were several differences in the dentition and oral papillae and fimbriae. In addition, the edges of the eyes, fins and over the pineal gland possess an iridescent blue/green coloration only in *G*. *macrostoma*. Similar to *G*. *australis*, *G*. *macrostoma* is also characterized by significant developmental changes through its spawning run, and by sexual maturity the growth of the oral disc exceeded that recorded in any lamprey species. Presently, many anadromous lamprey species are threatened or in decline from a number of anthropogenic pressures. Currently, all ecological knowledge of *G*. *australis* is based on Australasian populations, which may not be applicable to *G*. *macrostoma*. To ensure the conservation and protection of the Patagonian lamprey further investigations are needed to understand its life history, biology and ecology throughout its range.
